# Injury Surveillance during a European Touch Rugby Championship

**DOI:** 10.3390/sports7030071

**Published:** 2019-03-21

**Authors:** Emma Cropper, Cari M. Thorpe, Simon Roberts, Craig Twist

**Affiliations:** 1 Department of Human and Health Sciences, The University of Huddersfield, Queensgate, Huddersfield HD1 3DH, UK; 2 Department of Health Professions, Manchester Metropolitan University, Brooks Building, Bonsall Street, Manchester M15 6AN, UK; M.Thorpe@mmu.ac.uk; 3 Department for Health, The University of Bath, Claverton Down, Bath BA2 7AY, UK; S.P.Roberts@bath.ac.uk; 4 Department of Sport and Exercise Sciences, The University of Chester, Parkgate Road, Chester CH1 4BJ, UK; c.twist@chester.ac.uk

**Keywords:** team-sport, fatigue, injury

## Abstract

Background: Touch (rugby/football) is a minimal contact sport for which the type and incidence of injuries remains unknown in Europe. Objectives: To establish the incidence, severity and nature of injuries sustained during a four-day European Touch Championship competition. Methods: A prospective cohort design was adopted to record match-related injuries during the European Touch Championships 2016. Injuries were collected from five countries and classified using the Orchard Sports Injury Classification (OSICS-10). Data were combined from all participating countries and injury incidence was recorded as number of injuries/1000 player hours. Results: A total of 135 injuries were recorded during the tournament with injury incidence calculated as 103.5 injuries per 1000 player match hours. Injuries were mainly recorded as transient (76%) occurring most frequently in the lower limb (69%). Injuries occurred more frequently on successive days, with exception to the final day of the tournament. The number of injuries was not different between the first and second half of matches and there was no relationship between the day of the tournament and the half of the match that injury occurred. Conclusion: Match injury incidence was 103.5 injuries per 100 player match hours. The most injured site was that of the lower limb, with the most common injury type reported as muscle/tendon injury. It is postulated that fatigue plays a role in injury incidence during a multiday tournament.

## 1. Introduction

Touch (rugby/football) is a minimal contact sport originating in the southern hemisphere, originally developed as a training regime for rugby league players. In 1998, the Australian Touch Association reported more than 200,000 registered players [1]. In the last 10 years, the sport has expanded globally and now has annual National and International competitions with the Touch World Cup taking place every four years [2]. The number of registered players in affiliated teams in the U.K. reached 20,000 in November 2014 [2].

In Touch, there are two teams of six players on the pitch at one time, with another eight players used as ‘rolling’ substitutes. International rules stipulate that a match lasts for 40 min over two 20-min halves. Each team has the opportunity to have six touches until the ball is handed over to the opposing team [3]. There is no tackling, scrummaging or kicking of the ball in Touch as occurs in other codes of rugby. Moreover, Touch is unique to other team sports, in that players compete in both single and mixed-sex competitions, which potentially imposes different demands on players depending on the category in which they compete. For example, international and regional male Touch players cover relative distances during a match of ~140 and ~125 m/min, respectively [4], which are partly explained by greater high-intensity running distances for international compared to regional players (~40 cf. ~25 m/min) [4]. International players are therefore exposed to greater external loads, which are also greater than values reported for contact versions of rugby [5,6,7].

The removal of collisions should mean that Touch has a lower incidence of injuries compared to the ~40–60% and ~80% of total match-related injuries from these mechanisms observed in rugby league [8,9] and rugby union [10], respectively. Data from the southern hemisphere [11] reported that the most common injuries sustained during competitive Touch matches were to the ankle (23%) and knee (12%), followed by injuries to the hand (19%) and shoulder (12%). Neumann et al. also reported that 71% of the injuries in Touch were to the lower limb, which is comparable to both codes of rugby across all standards, where the highest number of injuries occurs to the lower limb than to other body region [9,10,12].

National and international Touch matches are played in a tournament style comprising several matches in succession. Where several matches are played on the same day, or successive days, players are likely to be exposed to activities that increase fatigue and reduce performance capability [13,14,15]. Accordingly, the increased physical loads accompanied by short recovery time when playing several matches per day might directly influence the injury incidence reported in Touch players compared to other sports.

Touch’s status as an emerging sport in Europe means there are currently no published data describing the type and incidence of injuries sustained by European Touch. As Touch is an amateur sport, players selected to play at the ‘elite’ standard do so alongside their normal employment or academic study and self-fund their participation. If players are injured and unable to work, there are likely to be financial implications for these players. Accordingly, a duty of care exists from the sport’s governing bodies to identify injury patterns and mechanisms in order to implement appropriate injury prevention strategies and to minimise the risk of injury to players. Successful injury prevention programmes are based on accurate epidemiological data to develop the content of such programmes [16,17], and presently the majority of sports injury surveillance systems exist in elite rather than amateur sport settings [18]. Accordingly, this study sought to establish the incidence, severity and nature of injuries sustained during a four-day European Touch Championship Competition using a modified community-level rugby union injury surveillance form [19]. The resulting knowledge will support development of an evidence-based injury prevention and safety promotion programme in Touch rugby that, as yet, does not exist.

## 2. Materials and Methods

### 2.1. Participants

This study adopted a prospective cohort design to record match-related injuries during the four-day European Touch Championships 2016. In a tournament comprising 14 countries and 53 teams, injury data were collected on players representing five countries that volunteered to participate in the study, all of whom were in the top five ranked European countries. The five countries provided a total of 35 teams, comprising 361 male and 178 female players. The Executive Boards of all countries who were Federation of International Touch recognised were contacted by the researcher before the tournament and invited to take part in the study. Written consent was provided by all the Executive Boards of participating countries for their injury data to be recorded. Individual player consent was not gained as no identifying personal details were recorded for players other than sex and age. Institutional ethics approval (FREC reference: 054/16/EK/SES) for the study was granted from the University of Chester’s Faculty of Science and Engineering Ethics Committee. All work conformed to the recommendations of the Declaration of Helsinki.

Nine grass-covered fields were used in the tournament, all located on the same sports complex. All pool matches took place on Days 1–3 with teams playing 2–3 matches per day during this period. Finals and placement matches were played on Day 4 with a mean of two matches played on this day. All matches were 40 min in duration (2 × 20 min halves), unless a draw occurred in a match that required a result for placement or progression. In this instance, a ‘drop off’ was played whereby each team removes one player every 2 min down to a maximum of three players until a winning team is established.

Data quality in sports injury surveillance relies on accurate injury diagnosis and coding by the data collectors. In some cases, errors can be attributed to the background and expertise of the coders [20]. For the purpose of the study, all Executive Boards were asked to identify a suitably qualified medical person (QMP) supporting the teams during the duration of the tournament to complete the injury data forms. These QMPs were required to be educated to degree level as a physiotherapist or sports therapist in order to ensure prerequisite anatomical knowledge and diagnostic skills to standardise the data collection. These identified QMPs were contacted by the researcher before the tournament to explain the research and provide them with an information pack that included data collection forms, injury definitions and injury classifications for standardization. The researcher was also on hand throughout the tournament to help with the completion of the injury data forms and to collect any completed forms twice daily. Only injuries sustained during the tournament were documented and included in the study.

### 2.2. Injury Report Form

Injury surveillance in an amateur setting poses several challenges. Recruiting non-transient qualified volunteers who were able and willing to collect the data is an issue in a non-professional setting; as well as the financial implications of implementing the technology for accurate injury surveillance [21]. There are also issues with injury recall from both players and surveillance staff and the possibility of duplicating information between paper records and online injury surveillance systems [21]. Given the issues associated with injury surveillance in amateur sport settings [16,21], a paper surveillance system was employed to aid compliance by medical staff and allow instant completion that minimised the need for recall. This injury report form was a modified version of the form utilised by Roberts [10] to establish injuries in community-level rugby union. Some of the reported errors in injury surveillance data are related to a lack of consistency in both injury definitions and poor categorising of injuries [18]. Accordingly, the consensus statement on injury definitions in rugby union modified for Touch was used [22]. An injury was defined as “any physical complaint which was caused by a transfer of energy that exceeded the body’s ability to maintain its structural and/or functional integrity that was sustained by a player during a touch match or training irrespective of need for medical attention or time loss from touch activities.” [22] (pp. 329).

Previous studies looking into time loss injuries have considered both training and match time loss and have considered loss of play during a typical season [8,15,23,24,25]. However, due to the nature of the sport and Touch National leagues consisting of four multi-day tournaments distributed over several months, it is difficult to quantify the time loss in regards to both training and match play. For the purpose of this study, a ‘time-loss’ injury was therefore defined as an injury where a player was unable to take part in a match, or part of a match, during the tournament after the injury diagnosis.

For all recorded injuries, information was documented regarding player age, sex and position (i.e., middle, link or winger), the day of the tournament, match number and match half that the injury occurred. Injury was classified using the Orchard Sports Injury Classification (OSICS-10) to generate a four-code classification [26]. Using this coding system, injury diagnosis was specifically classified including anatomical site and general pathology [27]. The OSICS-10 has demonstrated a moderate level of inter-rater reliability that was deemed appropriate for this study, relying on a number of different clinicians to record injury data [27]. The use of this standardised classification system also enabled comparison with other injury surveillance studies. Injuries were classified as ‘transient’ if the player missed no matches or part of a match due to the injury, ‘minor’ if they missed one match or part of match, ‘moderate’ if they missed between two and four matches or part of the matches and ‘major’ if they missed five or more matches or parts of matches in order to allow comparison with other injury surveillance studies [28].

### 2.3. Data Analysis

Data were combined from all participating countries. Injury incidence was recorded as the number of injuries/1000 player hours of match exposure. Total player hours were calculated based on the number of divisions entered by a participating country and the number of matches played in the division and considered the number of players on the field and the length of the matches as per the methods previously described by Roberts [10]. Ninety-five percent (95%) confidence intervals (CIs) were calculated using Poisson distribution [10]. Daily match number was not considered, as the tournament schedule involved a variation in the number of matches being played between each squad both per day, and the total amount of matches played over the duration of the tournament. The timing of the injuries reported was analysed in terms of injury occurrence per day and the match half that the injury occurred. Chi-squared (χ^2^) tests were used to identify any differences, with significance accepted if the *p* value was <0.05.

## 3. Results

### 3.1. Overall Incidence and Severity

A total of 135 injuries were recorded during the tournament, of which 32 and 103 were reported as occurring in female and male athletes, respectively (χ^2^ = 37.34, *p* < 0.05), which accounted for 29% of men compared to 18% of females being injured. The age range of injured athletes was 16–58 years (mean age 34; 95% CI: 32–35). The match injury incidence was 103.5 injuries per 1000 player match hours (95% CI: 86.1–121.0). The severity of injuries was mainly recorded as transient 76% (n = 102) with 14% (n = 19) of injuries recorded as minor, 8% (n = 11) as moderate and 2% (n = 3) as major. When considering injury by player position, the most injuries occurred in middles (n = 63, 47%) followed by links (n = 46, 34%) and then wingers (n = 26, 19%) (χ^2^ = 15.24, *p* < 0.05).

### 3.2. Injury Site and Type

Injury incidence was different between sites (χ^2^ = 65.0, *p* < 0.05; Table 1), with the lower limb (n = 93; 69%) higher compared with injuries to the head/trunk/upper limb (n = 39; 29%). More specifically, the most injured site was that of the hip/groin/thigh region (27%), followed by the knee/anterior lower leg (21%) and calf/Achilles/ankle/foot (21%). In addition, 2% (n = 3) of injuries were not recorded as being related to a body type and were systemic in nature. The most common injury type reported was muscle/tendon injures (n = 45; 33%) occurring most frequently in the lower limb (92% of reported muscle injuries). These were closely followed by contusion/laceration (n = 43; 32%), which were also most frequently reported as occurring in the lower limb (74% cf. 26% in the upper limb).

### 3.3. Timing of Injuries

**Figure 1:**
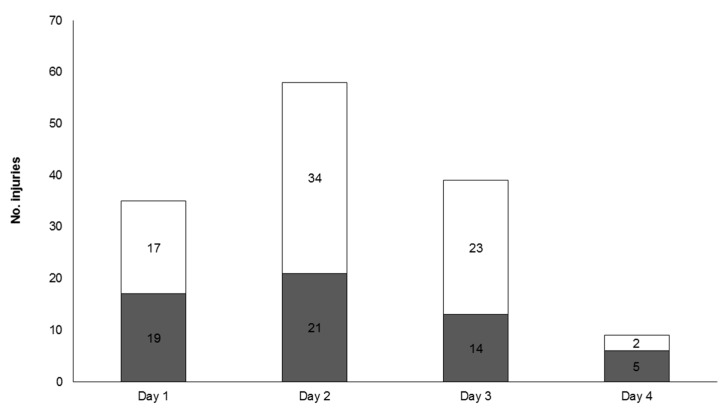
Total injuries per day with number of first (■) and second (□) half match injuries.

Injuries were significantly different between days of the tournament (χ^2^ = 35.04, *p* < 0.05), with most injuries occurring on Day 2 (n = 55, 41%), followed by Day 3 (n = 37, 27%), Day 1 (n = 36, 27%) and then Day 4 of the tournament (n = 7, 5%). More injuries were sustained during the second half of matches (n = 78, 58%) compared to the first half (n = 57, 42%; Figure 1); however, this was not significant (χ^2^ = 2.14, *p* > 0.05; Table 2). There was also no relationship between the day of the tournament and the half of the match that injury occurred (χ^2^ = 4.591, *p* > 0.05).

### 3.4. Mechanism of Injury

The most common mechanism of injury reported was during running-based activities (41%), closely followed by diving-based (26%) and collision-based (26%) activities. The greatest proportion of injuries sustained from running activities were muscle and tendon injuries (27%), and those sustained from diving- and collision-based activities were contusion/laceration injuries (30%). The mechanism of injury and injury type is illustrated in Table 3.

## 4. Discussion

To the authors’ knowledge, this is the first study to examine the injury incidence, type and impact for elite amateur touch players during a competitive tournament in Europe. This study has identified that: (i) injury incidence is 103.5 injuries/1000 h player-match exposure during an international Touch tournament; (ii) most injuries are muscle/tendon injuries to the lower extremities, namely hamstring and calf, respectively; and (iii) injuries predominately occurred during the second day of the four-day tournament.

The overall injury incidence for elite Touch players during a tournament was 103.5 injuries per 1000 player-match hours, which is similar to the 108.3 injuries per 1000 player-match hours reported for elite Rugby-7s players during tournament match play [15]. However, the injury incidence for Touch is much higher than that reported from the International Rugby Board Rugby World Cup 2011 and 2015, where the overall injury incidence was 89.1 and 90.1 injuries per 1000 player-match hours, respectively [25,29]. Our data also compare less favorably to elite rugby league injury incidence values of 40.3 injuries per 1000 player-match hours from a pooled data analysis [30]. A higher injury incidence in Touch is probably explained by several contributing factors. Firstly, some differences might have occurred due to the use of a modified surveillance form. Also, Touch remains an amateur game that means there is a low frequency of contact between the players and coaching/medical staff. Training is typically limited to once per month, with players required to follow prescribed, but unsupervised training schedules remotely. Players’ compliance with unsupervised training practices is likely to be variable [31], which leads to greater variations in skill and physical qualities compared to those found in professional full-time players [10]. Greater running demands than other rugby codes [4] because of the absence of collisions [32], and that Touch players play multiple matches over several days with limited recovery, means that inadequacies in physical qualities and skill could expose Touch players to a greater risk of injury [10,33]. A higher mean age reported for players in this study (34 years; 95% CI: 32–35 years) is also likely to increase the risk of injury in Touch players further, given the known age-related decline in physical qualities occurring from 35 years of age [34,35,36]. In addition, it is likely that older players will have increased commitments outside of the sport, including work and family responsibilities, which might affect the time they are able to dedicate to training. This could cause a further decrease in fitness and ultimately increase the injury risk in older players.

There was a much greater proportion of transient injuries (76%) reported in Touch compared to other codes of rugby [19,25,29,30]. This resulted in less time loss injuries compared to other codes [19,25,29,30] that might be accounted for by a narrower definition of injury between studies and the exclusion of non-time loss injuries in several studies [19,25,29]. Furthermore, injured Touch players might continue to play despite medical advice due to the limited opportunities to play at an international standard. As players are self-funding, they could be less likely to disclose the true extent of injuries with concern of being removed from play.

Injury location for Touch players occurred predominantly in the lower limb. These findings are consistent with those previously reported in Australian Touch players [1] and suggest that the lower limb remains a key area for injury prevention in this group of athletes. The most prevalent type of injury reported in this study was muscle/tendon injuries (33%), of which the majority were attributed to running-related activities (41%). These observations are similar to data reported previously in other codes of rugby [37,38,39,40], particularly for non-contact injury [19]. The high prevalence of muscle injuries in Touch, particularly in the lower limb, could be attributable to a combination of environmental and individual factors, as well as touch-specific mechanisms.

Like rugby-7s [5], Touch players are required to repeat short-duration, high-intensity sprints during a match [4]. In sports involving sprinting, hamstring injuries are particularly prevalent [41,42,43,44,45,46,47]. Causative factors in hamstring strains are numerous, including: hamstring muscle weakness, poor hamstring flexibility and muscle fatigue [47]. The ‘dump and scoop’ action by which the ball is played after a ‘touch’ is unique in that it requires deceleration, forward flexion of the trunk to place the ball on the floor, followed by acceleration to follow the line of play. The required flexibility of the hamstrings during this movement pattern and the associated fatigue from high-intensity running efforts [5] could be a causative factor in the number of hamstring strains identified in Touch. In addition, ground conditions might be a risk factor in injury incidence [48]. Touch is a ‘summer sport’, and international tournaments take place between July and August on grass pitches. However, training typically starts in the winter when training surfaces are likely to be softer. Improved environmental conditions as the year progresses result in harder playing surfaces, enabling players to reach higher running speeds but with decreased shock absorption from the ground [38,39,49]. It has been reported that soft tissue injuries, including muscular strains, were the most commonly occurring injuries in grounds with greater hardness [50], which is related to the effect the surface has on absorbing impact energy and the traction a playing surface provides [48]. Accordingly, discrepancies between training and competition playing surfaces for Touch players might explain the high incidence of lower limb muscle strains reported in this study (33%). Harder, drier ground will also enable greater peak reaction forces than softer ground when a player lands [50] that could provide the mechanism to explain the high incidence of diving/collision (52% in total) and contusion/laceration (33%) type injuries.

International Touch tournaments comprise multiple matches played over successive days with limited recovery. Similar competition schedules in international Tag football (a variant of Touch) have been shown to cumulatively increase feelings of fatigue and muscle soreness [14]. Movement characteristics of elite rugby-7s players during a multiday tournament have also been shown to reduce in the second half of matches, including a reduction in the total distance covered per minute and a decline in the moderate- and high-speed accelerations per minute [5]. Other studies [15] also identified that after five matches in a tournament setting there was a 37% probability that Rugby-7s players would have sustained an injury. Fatigued muscles’ loss of coordination and reduced ability to absorb energy has been proposed to increase the risk of injury during rugby match play [5,15]. Therefore, it is possible that Touch players exposed to multiple matches within a tournament will experience cumulative fatigue and an increased injury incidence in the latter stages.

The acute:chronic workload ratio is known to influence injury risk in team sport players [51]. Acute workload is defined as the workload performed in one week relative to chronic workload defined as the four week mean workload [52]. Touch is played on an amateur basis and with no jurisdiction on training regimes in terms of hours to be dedicated to training alongside players’ other outside commitments. In a tournament setting, players are exposed to a ‘spike’ in their workloads, where training and playing load probably increase above their mean loads over the previous four-week period [51]. This might predispose athletes to an increase in injury risk if their training is not sufficient in the lead up to a tournament.

Interestingly, this study observed that most of the injuries occurred on Day 2 of the tournament, with the least injuries occurring on the last day. This could be due to the effects of an initial spike in acute workload on Day 1 compared to what the players were typically accustomed to [52]. Players therefore enter the second day of the tournament in a more fatigued state, thus increasing injury risk on Day 2 [15]. In addition, the number of reported injuries might decrease as players injured earlier in the tournament are removed from play in subsequent days, and a reduction in player numbers involved in finals on Day 4 of the tournament as teams were knocked out of the competition.

Touch is unique in its mixed sex participation. Interestingly, in this study, females reported less injuries than males. It has previously been documented that inferior physical qualities and lower skill ability in females compared to males could account for differences in reported injury incidence [53]. There were, however, less opportunities for females to play in a squad during the tournament. Of the nine categories, there were only four in which women could participate: two open squads (women’s open and mixed open) and two age-restricted squads (senior mixed and women’s 27 s). In mixed-sex teams, females tend to play in a wing position, which in this study reported the least number of injuries (19%). Similar playing positions in Tag football have been shown to possess a greater lower body muscular power and straight line running speed than inside players. In addition, compared to inside players, outside players have greater chance to recover between repeated very-high-speed running efforts [14]. Accordingly, a tendency for female players to be selected to play in less physically demanding positions in mixed-sex teams might reduce their exposure to increased injury risk. In addition, previous research has identified an increased physical demand of players in the middle position, which might be explained by the increased involvement in both attacking and defensive patterns [54]. This supports our study’s findings that there were more injuries reported in middle players compared to other playing positions. This is something that should be considered for future research.

## 5. Limitations

The authors acknowledge several limitations with this study. Given the scale of the data collection and use of multiple countries, it was necessary to use different staff to complete the injury surveillance report forms. The challenges of injury reporting across several teams have been identified in other injury surveillance studies and include staff time, constraints during the tournament alongside providing medical support, differences in expertise for medical diagnosis, interest in the data and language barriers [17,32]. However, the use of medical professionals with a minimum qualification to report injuries and the use of a standardised data collection form and coding system will have improved inter-rater reliability and the quality of the collected data [27]. While not reported, we also observed similar injury patterns across the different countries in terms of site, type, mechanism and time loss. The use of only five countries was caused by other nations not having a suitably qualified medical practitioner or unwillingness to participate. Finally, this study concentrated on elite international players during a four-day tournament, which means the findings cannot be generalised to those players competing at lower standards. Further research should concentrate on injury surveillance in community-level competition to enable implementation of appropriate preparation and management strategies in this group.

## 6. Conclusions

For the first time, this study provides information on the injury incidence, type and impact for elite amateur touch players in Europe. Whilst there is a perceived reduction in injury risk in Touch compared to other rugby codes due to the removal of physical contact, this study reported a higher injury incidence than observed in elite rugby union and league but a comparable injury incidence to Rugby-7s. Most reported injuries were transient in nature resulting in no loss of play and the most common injury was to muscle/tendon injury in the lower limb from running-type activities. We recommend specific strength and conditioning programmes and effective rehabilitation programmes in order to ensure that Touch players are physically prepared for tournament competition. In particular, the ability to withstand fatigue-related injury appears to be important and as a consequence conditioning programmes should be designed to minimise these effects by promoting appropriate adaptations. For example, training drills that enable players to be progressively exposed to performing fundamental movements and skills under fatigued conditions should be incorporated into pre-competition training programmes. Due to the high number of reported injuries, this study also highlights the need for adequate medical support in a tournament setting and the injury management skills. Coaches and those responsible for the governance of Touch should acknowledge the findings of this study, particularly given its amateur status where injuries could impact on a player’s commitments outside of the sport, including employment.

## Figures and Tables

**Table 1:** Injuries according to location and type.

Location and Type		Number of Injuries	
		Total	Percentage of Total Injuries (%)
Head/neck/face	Laceration	3	2%
	Whiplash	2	1%
	Head injury/concussion	6	4%
	Jaw	1	0.70%
	Total	12	
Shoulder/arm/elbow	Shoulder strain	3	2%
	Bruising/Haematoma	5	4%
	Laceration	3	2%
	Nerve injury	2	1%
	Total	13	
Forearm/wrist/hand	Thumb/finger sprain	6	4%
	Fracture (metacarpals)	2	1%
	Blood injury	1	0.7%
	Flexor tendon injury	1	0.7%
	Total	10	
Trunk/spine	Lumbar facet strain	2	1%
	Thoracic pain	1	0.7%
	Rib bruising	1	0.7%
	Total	4	
Hip/groin/thigh	Laceration	5	4%
	Bruising/haematoma	11	8%
	Muscle strain	21	15%
	Total	37	
Knee/anterior lower leg	Laceration	8	6%
	Bruising/haematoma	2	1%
	Shin splints	4	3%
	Meniscal injury	3	2%
	Soft tissue knee injury	6	4%
	ACL rupture	1	0.7%
	Undiagnosed knee pain	2	1%
	Patella tendinopathy	2	1%
	Total	28	
Calf/Achilles/ankle/foot	Blister/black toe nail	3	2%
	Mid-foot/hallux sprain	2	1%
	Muscle strain	13	10%
	Ankle ligament strain	4	3%
	Ankle impingement	3	2%
	Plantar fasciitis	2	1%
	Ankle fracture	1	0.7%
	Total	28	
Miscellaneous	Heat-related illness	3	2%
All Injuries		135	100%

ACL = anterior cruciate ligament.

**Table 2:** Injury type and timing with respect to first and second half match.

Mechanism/Injury Type	No. Injuries	No. Injuries (%)	Timing of Injury	
			First Half	Second Half
Fracture and bone stress	10	7	4	6
Joint/ligament injury	21	16	9	12
Muscle/tendon injury	45	33	17	28
Contusion/laceration	43	32	25	18
Nerve/neural injury	8	6	2	6
Undiagnosed/systemic	8	6	0	8

**Table 3:** Associations between mechanism and injury type.

Mechanism/Injury Type	Running	Passing	Catching	Diving	Collision	Stretching
Fracture and bone stress	5			1	4	
joint/ligament injury	7	1	2	5	7	1
muscle/tendon injury	36	1	1	4	3	
contusion/laceration	3			22	18	
nerve/neural injury				2	6	
undiagnosed/systemic	3					
Total	56	2	3	35	35	1

## References

[1] Neumann D.C., McCurdle I.M., Wade A.J. (1998). A survey of injuries sustained in the game of touch. J. Sci. Med. Sport.

[2] England Touch Association. www.englandtouch.org.uk.

[3] Federation of International Touch. https://www.internationaltouch.org/.

[4] Beaven R., Highton J., Thorpe C., Knott E., Twist C. (2014). The movement and physiological demands of international and regional men’s touch rugby matches. J. Strength Cond. Res..

[5] Higham D.G., Pyne D.B., Anson J.M., Eddy A. (2011). Movement patterns in rugby sevens: Effects of tournament level, fatigue and substitute players. J. Sci. Med. Sport.

[6] Waldron M., Twist C., Highton J., Worsfold P., Daniels M. (2011). Movement and physiological match demands of elite rugby league using portable global positioning systems. J. Sports Sci..

[7] Cahill N., Lamb K., Worsfold P., Headey R., Murray S. (2013). The movement characteristics of English Premiership rugby union players. J. Sports Sci..

[8] Fitzpatrick A.C., Naylor A.S., Myler P., Robertson C. (2018). A three-year epidemiological prospective cohort study of rugby league match injuries from the European Super League. J. Sci Med Sports.

[9] King D.A., Gabbett T.J., Dreyer C., Gerrard D.F. (2006). Incidence of injuries in the New Zealand national rugby league sevens tournament. J. Sci. Med. Sport.

[10] Roberts S.P., Trewartha G., England M., Shaddick G., Stokes K.A. (2013). Epidemiology of time-loss injuries in English community-level rugby union. BMJ Open..

[11] Hockney R., Miles E. (1997). Injury Bulletin, Football Injuries.

[12] Williams S., Trewartha G., Kemp S., Stokes K. (2013). A meta-analysis of injuries in senior men’s professional Rugby Union. Sports Med..

[13] Johnston R., Gibson N.V., Twist C., Gabbett T.J., MacNay S., MacFarlane N. (2013). Physiological responses to an intensified period of rugby league competition. J. Strength Cond. Res..

[14] Hogarth L.W., Burkett B.J., McKean M.R. (2015). Neuromuscular and perceptual fatigue responses to consecutive tag football matches. Int. J. Sports Physiol. Perform..

[15] Fuller C.W., Taylor A.E., Raftery M. (2016). Should player fatigue be the focus of injury prevention strategies for international rugby sevens tournaments?. Br. J. Sports Med..

[16] Ekegren C.L., Gabbe B.J., Donaldson A., Cook J., Llyod D., Finch C.F. (2015). Injuries in community-level Australian football: Results from a club-based injury surveillance system. J. Sci. Med. Sport.

[17] Ekegren C.L., Gabbe B.J., Finch C.F. (2015). Injury surveillance in community sport: Can we obtain valid data from sports trainers?. Scandinavian J. Med. Sci. Sports.

[18] Ekegren C.L., Gabbe B.J., Finch C.F. (2016). Sports Injury Surveillance Systems: A Review of Methods and Data Quality. Sports Med..

[19] Roberts S.P., Trewartha G., England M., Stokes K. (2014). Incidence and nature of medical attendance injuries in English community rugby union. Orthop. J. Sports Med..

[20] Finch C.F., Orchard J.W., Twomey D.M., Saad Saleem M., Ekegren C.L., Lloyd D.G., Elliott B.C. (2014). Coding OSICS sports injury diagnoses in epidemiological studies: Does the background of the coder matter?. Br. J. Sports Med..

[21] Ekegren C.L., Donaldson A., Gabbe B.J., Finch C.F. (2014). Implementing injury surveillance systems alongside injury prevention programs: Evaluation of an online surveillance system in a community setting. Inj. Epidemiol..

[22] Fuller C.W., Molloy M.G., Bagate C., Bahr R., Brooks J.H.M., Donson H., Kemp S.P.T., McCrory P., McIntosh A.S., Meeuwisse W.H. (2007). Consensus statement on injury definitions and data collection procedures for studies of injuries in rugby union. Br. J. Sports Med..

[23] McNoe B.M., Chalmers D.J. (2010). Injury in Community-Level Soccer: Development of an Injury Surveillance System. Am. J. Sports Med..

[24] Schwellnus M.P., Thomson A., Derman W., Jordaan E., Readhead C., Collins R., Morris I., Strauss O., Van der Linde E., Williams A. (2014). More than 50% of players sustained a time-loss injury (>1 day of lost training or playing time) during the 2012 Super Rugby Union Tournament: A prospective cohort study of 17,340 player-hours. Br. J. Sports Med..

[25] Fuller C.W., Sheerin K., Targett S. (2013). Rugby World Cup 2011: International Rugby Board Injury Surveillance Study. Br. J. Sports Med..

[26] OSICS. http://www.johnorchard.com/about-osics.html.

[27] Hammond L.E., Lilley J., Ribbans W.J. (2016). Coding sports injury surveillance data: Has version 10 of the Orchard Sports Injury Classification System improved the classification of sports medicine diagnoses?. Br. J. Sports Med..

[28] King D.A., Gabbett T.J., Gissane C., Hodgson L. (2009). Epidemiological studies of injuries in rugby league: Suggestions for definitions, data collection and reporting methods. J. Sci. Med. Sport.

[29] Fuller C.W., Taylor A., Kemp S.P.T., Raftery M. (2017). Rugby World Cup 2015: World Rugby injury surveillance study. Br. J. Sports Med..

[30] Gissane C., Jennings D., Kerr K., White J. (2002). A Pooled data analysis of injury incidence in rugby league football. Sports Med..

[31] Kovacs M.S., Pritchett R., Wickwire P.J., Green J.M., Bishop P. (2007). Physical performance changes after unsupervised training during the autumn/spring semester break in competitive tennis players. Br. J. Sports Med..

[32] Roberts S.P., Trewartha G., England M., Stokes K. (2015). Collapsed scrums and collision tackles: What is the injury risk?. Br. J. Sports Med..

[33] Gabbett T.J., Donrow N. (2007). Relationships between training load, injury, and fitness in sub-elite collision sport athletes. J. Sports Sci..

[34] Gent D.N., Norton K. (2012). Aging has greater impact on anaerobic versus aerobic power in trained masters athletes. J. Sports Sci..

[35] Reaburn P., Dascombe B. (2008). Endurance performance in masters athletes. Eur. Rev. Aging Phys. Act..

[36] Tanaka H., Seals D.R. (2008). Endurance exercise performance in Masters athletes: Age-associated changes and underlying physiological mechanisms. J. Physiol..

[37] Gabbett T.J. (2000). Incidence, site, and nature of injuries in amateur rugby league over three consecutive seasons. Br. J. Sports Med..

[38] Gissane C., Jennings D., White J., Cumine A. (1998). Injury in summer rugby league football: The experiences of one club. Br. J. Sports Med..

[39] Gissane C., Jennings D., Kerr K., White J. (2003). Injury rates in rugby league football: Impact of change in playing season. Am. J. Sports Med..

[40] King D.A., Gabbett T.J. (2008). Training injuries in New Zealand amateur rugby league players. J. Sci. Med. Sport.

[41] Brooks J.H., Fuller C.W., Kemp S.P., Reddin D.B. (2005). Epidemiology of injuries in English professional rugby union: Part 1 match injuries. Br. J. Sports Med..

[42] Brooks J.H., Fuller C.W., Kemp S.P., Reddin D.B. (2005). Epidemiology of injuries in English professional rugby union: Part 2 training injuries. Br. J. Sports Med..

[43] Brooks J.H., Fuller C.W., Kemp S.P., Reddin D.B. (2006). Incidence, risk, and prevention of hamstring muscle injuries in professional rugby union. Am. J. Sports Med..

[44] Croisier J.L. (2004). Factors associated with recurrent hamstring injuries. Sports Med..

[45] Petersen J., Holmich P. (2005). Evidence based prevention of hamstring injuries in sport. Br. J. Sports Med..

[46] Woods C., Hawkins R.D., Maltby S., Hulse M., Thomas A., Hodson A. (2004). The football association medical research programme: An audit of injuries in professional football—Analysis of hamstring injuries. Br. J. Sports Med..

[47] Yeung S.S., Suen A.M.Y., Yeung E.W. (2009). A prospective cohort study of hamstring injuries in competitive sprinters: Preseason muscle imbalance as a possible risk factor. Br. J. Sports Med..

[48] Petrass L.A., Twomey D.M. (2013). The relationship between ground conditions and injury: What level of evidence do we have?. J. Sci. Med..

[49] Hodgson-Phillips L., Standen M.E., Batt M.E. (1998). Effects of seasonal change in rugby league on the incidence of injury. Br. J. Sports Med..

[50] Twomey D.M., Finch C.F., Llyod D.G., Elliott B.C., Doyle T.L.A. (2012). Ground hardness and injury in community level Australian football. J. Sci. Med. Sport.

[51] Hulin B.T., Gabbett T.J., Caputi P., Lawson D.W., Sampson J.A. (2016). Low chronic workload and the acute:chronic workload ratio are more predictive of injury than between-match recovery time: A two-season prospective cohort study in elite rugby league players. Br. J. Sports Med..

[52] Blanch P., Gabbett T.J. (2014). Has the athlete trained enough to return to play safely? The acute:chronic workload ratio permits clinicians to quantify a player’s risk of subsequent injury. Br. J. Sports Med..

[53] Hagglund M., Walden M., Ekstrand J. (2009). Injuries among male and female elite football players. Scand. J. Med. Sci. Sports.

[54] Vickery W., Harkness A. (2017). Physical, Physiological and perceptual match demands of amateur mixed gender touch players. J. Sports Sci. Med..

